# Automated Vibroacoustic Monitoring of Trees for Borer Infestation

**DOI:** 10.3390/s24103074

**Published:** 2024-05-12

**Authors:** Ilyas Potamitis, Iraklis Rigakis

**Affiliations:** 1 Department of Music Technology & Acoustics, Hellenic Mediterranean University, 741 33 Rethymno, Greece; 2 Department of Electronics, Hellenic Mediterranean University, 731 33 Chania, Greece; rigakis@hmu.gr; 3 INSECTRONICS, 73100 Chania, Greece

**Keywords:** woodboring insects, biodiversity, sensors, signal processing, vibrations, AI, IoT, invasive pests

## Abstract

In previous research, we presented an apparatus designed for comprehensive and systematic surveillance of trees against borers. This apparatus entailed the insertion of an uncoated waveguide into the tree trunk, enabling the transmission of micro-vibrations generated by moving or digging larvae to a piezoelectric probe. Subsequent recordings were then transmitted at predetermined intervals to a server, where analysis was conducted manually to assess the infestation status of the tree. However, this method is hampered by significant limitations when scaling to monitor thousands of trees across extensive spatial domains. In this study, we address this challenge by integrating signal processing techniques capable of distinguishing vibrations attributable to borers from those originating externally to the tree. Our primary innovation involves quantifying the impulses resulting from the fracturing of wood fibers due to borer activity. The device employs criteria such as impulse duration and a strategy of waiting for periods of relative quietness before commencing the counting of impulses. Additionally, we provide an annotated large-scale database comprising laboratory and field vibrational recordings, which will facilitate further advancements in this research domain.

## 1. Introduction

The longhorn beetles (Cerambycidae family) among many other xylophagus families of Arthropoda inflict extensive harm to both softwood and hardwood species within stored logs, forestry, and agriculture, resulting in significant financial ramifications, with annual losses estimated in the billions of euros. These invasive pests not only degrade timber resources but also disrupt ecosystem services, tourism, and biodiversity, exacerbating their detrimental impact on both the environment and the economy. The issue is compounded by climate change, and intervention efforts across the globe are currently fragmented [1]. 

Many wood-boring insect species are considered priority pests in the European Union, whose surveillance in non-infested areas as well as monitoring and control in all invaded countries is mandatory. National and regional governmental institutions in Europe oversee these activities but there is a lack of international coordination and sharing of data and management procedures. In this work, we introduce remotely controlled devices that can report automatically if a tree is infested with wood-boring insects after analyzing its internal vibrational content for a period of time. Making the device unattended is a key component to expand monitoring to thousands of strategically selected trees that are dispersed at large special scales. Automation carries significant implications as it generates continuous streams of data amenable to automatic interpretation by machine learning methodologies. Moreover, being able to monitor trees without human intervention allows to connect the stream of data from the trees with eco-epidemiological modelling, decision support systems (DSS), and artificial intelligence tools such as ChatGPT [2] to deliver networks of early warnings and potential for effective response to invasive woodboring insects at the European level.

Regardless of the intervention strategy implemented to mitigate the impact of invasive xylophagous insects, understanding the precise location and severity of the infestation, as well as monitoring the evolution of the phenomenon, is not only beneficial but often imperative. Visual assessment of infestation, such as the fall of leaves at the crown of palms, exit tunnels, and discoloration of leaves, typically occurs too late, by which time the infestation has often become uncontrollable. Remote automatic monitoring aims to detect the pest during the early stages of infestation and make informed decisions after monitoring the same tree remotely for an extended period, ranging from days to weeks.

This knowledge allows for the designation of infested areas, enabling the prioritization of actions (removal of heavily infested or dead trees, chemical or biological treatment of trees [3], and remote assessment of their efficacy) and allocation of funds to actions based on the severity of the issue. Several methods can be employed to analyze vibroacoustic recordings emanating from the interior of a living tree. The primary approach involves auditory examination, wherein human observers can readily discern the sequence of impulses associated with insect woodboring activity. Larvae at various instar levels dig tunnels under the bark or inside the tree and emerge from the tree as adults. The cracking of fibers is inevitable and produces a distinctive sound pattern characterized by a short duration and a broad frequency range. This vibrational signal associated with the latter activity gives in their existence and can be remotely uploaded to a remote server through IoT connectivity. Listeners, even untrained ones, can decide whether successive 30 s recordings have vibrational activity associated with infestation. However, a limitation of this method is that one must listen to the entire recording, as relevant evidence may emerge towards its end. Consequently, this auditory approach is not scalable to many recordings, typically in the order of hundreds. 

The utilization of vibroacoustic monitoring of trees for xylophagous insects has a long history [4], as examined in comprehensive reviews spanning a century of insect acoustic detection [5,6,7]. During this period, commercial products like the AED2000 employed a piezoelectric crystal and electronic circuitry to deliver audio output to headphones [8]. While deemed reliable in its time, a contemporary perspective would identify it as unwieldy, featuring large piezoelectric components adhered together, highly noisy, lacking wireless transmission capabilities, and devoid of local storage for recordings, among other drawbacks. Various authors proposed alternative research-oriented implementations of the vibroacoustic method [9,10]. However, in our previous work, we introduced a modern iteration of this traditional concept that incorporated numerous technological advancements available at that time [11]. Our updated design boasted compact dimensions, integrated energy harvesting for autonomous operation with minimal maintenance requirements, remote controllability, and utilized an advanced piezoelectric element approximately one-twentieth the size of the AED2000’s component. Furthermore, it featured wireless uploading of compressed vibrational recordings, employed low-power, low-noise electronics, and was housed in a weather-resistant enclosure suitable for field deployment. Nevertheless, human intervention remained essential in the decision-making process, as operators were still required to access the server and listen to the vibrational recordings to assess manually the infestation status of the trees. 

In this study, we introduce a streamlined, automated two-stage signal processing and sensor integration aimed at distinguishing between short-term peaks originating from borers and those stemming from external sources of vibrations. Subsequently, the procedure proceeds to tally the peaks associated with impulses from vibrating fractured fibers. This framework enables deployment across extensive temporal and spatial scales, facilitating a transition from indiscriminate chemical applications to consultancy and local intervention based on knowledge and insights.

The manuscript is organized in Section 2—Materials and Methods where Section 2.1 is about the hardware and Section 2.2. describes the signal processing approach that discerns and counts the impulses originating from borers. In Section 3—where Section 3.1 reports in-lab experiments with high-quality vibration data from which we derive duration thresholds that are applied in a series of field experiments reported in Section 3.2. Section 4—Discussion comments on the findings of Section 3 and projects into future applications.

## 2. Materials and Methods

**Figure 1:**
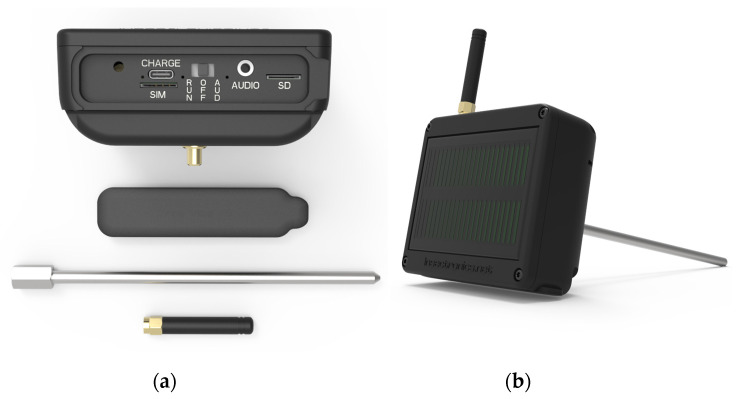
(**a**) The housing (10.1 cm × 8.3 cm × 4.3 cm) contains an electronic board and a communication antenna. A metal rod (the coupler between vibes and the electronics’ board) is inserted in the tree’s trunk. (**b**) The assembled device. The device has embedded energy harvesting through a solar panel in its back connected to its rechargeable battery.

The core component of the devices is the electronic board, which converts recorded vibrations into audio signals. These signals are compressed, stored, and wirelessly transmitted, along with metadata (refer to Figure 1). The metadata includes GPS coordinates, temperature, local timestamp, and counts of the impulses originating from xylophagous pests. Integrated within the board is a piezoelectric sensing element that converts the small deformations induced by micro-vibrations into voltage fluctuations. These voltage variations are then amplified and further processed by the low-power electronic board.

The device periodically samples the internal vibroscape of the tree, for example, every hour for 30 s, but this sampling rate can be remotely reconfigured as needed. The embedded code analyzes the recordings to identify impulsive events and then enters a sleep mode. At predetermined intervals as defined at the server level, it wakes up, primarily transmitting the metadata along with the option to transmit recordings, to the server via the mobile network or WiFi if available, before shutting down.

Micro vibrations within the tree are captured by an inserted waveguide, typically a stainless-steel bar that acts as a vibrations’ coupler between the wood and the sensing element. The waveguide comes in various sizes tailored to the dimensions of the tree. For instance, the trunk diameter of a mature *Phoenix canariensis* can vary significantly based on factors like age and environmental conditions. Typically ranging from 2 to 4 feet (0.6 to 1.2 m), or even larger at the base and/or crown, a 20 cm-long probe is suitable for such specimens. Conversely, a young fig tree may have a trunk diameter between 10 to 20 cm, necessitating a thinner and shorter metal rod akin to a small drill bit to avoid tree damage. Notably, the drill bit itself can serve as the probe, ensuring harmlessness to the tree.

It is crucial to recognize that an external microphone, used to sense audio from a distance without drilling or inserting a probe, is unable to detect these micro-vibrations except in rare cases where adult borers are in a developmental stage and are ready to emerge from the trunk, and the species is substantial. Conversely, surface sensors affixed to a properly prepared spot on the trunk (e.g., smoothed or bark-removed area, with stable sensor contact) may register some vibrations. However, there will be significant attenuation, resulting in the detection of only the strongest pulses—a mere fraction of the total existing pulses. This selective detection of strong pulses reduces the likelihood of detecting activity in early instar stages and instances of very low infestation.

The device, through the WiFi or mobile network, accesses the cloud server and uploads metadata to be visualized, and if requested by the user, the vibrational signals themselves as supportive evidence, so that the users have a permanent record to listen, catalogue, and share and optionally examine them manually through sonograms and spectrograms. Through the server, the device is configured and receives commands and new firmware. Commands include parameters such as the duration of the recording and the frequency of the reporting sessions. In this work, we present field trials of the pest *Xylotrechus chinensis* but the device has been successfully applied to the cases of *Rhynchophorus ferrugineus* (Red palm weevil), mountain bark beetle, emerald ash borer, *Rhynchophorus palmarum, Aromia bungii* (Red necked longicorn), *Anoplophora glabripennis* (Asian longhorn beetle), and *Anoplophora chinensis* (Citrus longhorn beetle) among many other xylophagous pests. 

### 2.1. Materials

Platforms designed for long-term deployment must be custom-made for the specific application and engineered to be low power to minimize maintenance requirements. The microprocessor carrying out all processing and communication with the submodules is the STM32L476RG (ST Microelectronics, Geneva, Switzerland). The STM32L476RG device is an ultra-low-power microcontroller based on the high-performance Arm Cortex-M4 32-bit RISC core operating at a frequency of up to 80 MHz. The device carries a GPS and a 4G-LTE SIM7600 (SIMCOM Shanghai, China), modem to transmit the recordings and the device’s position. Prior to transmission, the waveforms are compressed using the mp3 compression protocol. The board also has a WiFi functionality mainly for the examination of tree trunks in laboratory conditions (we use an ESP32 microcontroller for this). The waveguide is attached to an embeddable accelerometer 66333PNZ1 (PCB, Piezotronics, NY, USA), 3-wire low power, 1000 mV/g, TO8 housing, that is based on a stable piezo-ceramic crystal with an embedded low power amplifier. The frequency range (±3 dB) is 0.5 to 5000 Hz and has a resonant frequency >16 kHz. We chose this accelerometer mainly for its wideband frequency response where feeding impulsive sounds lie and its sensitivity at 1000 mV/g. The accelerometer is very small and embeddable. 

The output of the accelerometer is amplified and converted into digital signals with 16-bit resolution, utilizing sampling rates of 8, 16, and 32 kHz, facilitated by the external ADC PCM1820 (Texas Instruments, Dallas, TX, USA). We opted for the external ADC due to its favorable characteristics, including a low energy profile (113-dB SNR) and low power consumption (19.6 mW/channel at a 16-kHz sample rate). Furthermore, its design obviates the need for an anti-aliasing filter during sampling, affording us the flexibility to choose from multiple sampling rates. The digital words are sent to the microcontroller via the I2S interface. The electronic board is powered by two rechargeable A4 batteries INR18650 Li-ion 2500 mAh 3.7 V by Samsung that are also connected to an embedded 5 V/100 mW solar panel (see Figure 1b). Charging is managed by a BQ24075 Texas Instruments integrated Li-ion linear charger and system power path management device that targets space-limited portable applications. 

The sampling frequency, record duration, and other initialization parameters are read once from the SD card during powering on and are configurable through the server. The software is written in C language using the STMCubeIDE version 1.13.2. The programming of the flash memory was carried out using the ST-Link V3 programmer. The code initialization was done using the STM32CubeMX version 6.9.2-RC4 of ST. For programming the peripheral sub-components such as the SD and ADC, we made use of the STM32 HAL Drivers. The control signals and data transfers were implemented using the DMA controller of STM32L476. 

### 2.2. Methods

Our method for determining the infestation status of a tree involves identifying repetitive pulses of short duration, which serve as an indication of woodboring insect activity. We chose to focus on this characteristic because the fracturing of fibers is a common occurrence across all species of xylophagous insects, irrespective of their host trees. However, the spectral composition of the vibrations varies depending on the species of pests and the specific combination of wooden substrate, which can differ significantly across various regions of the world. In reality, pests can produce much more vibrations inside the trunk than that. For example, the larvae of *Anoplophora glabripennis* and *A. chinensis* produce different patterns of vibration when crawling, clearing cavities, and being frightened, and these different behavioral patterns are associated with vibrations’ modes that have their own unique characteristics in the time and frequency domains [7]. These vibrational patterns, in addition to the fracturing of fibers, constitute integral components of their presence. However, to date, we have not encountered a woodboring insect that does not produce fracturing sounds in addition to other sounds. Our methodology relies on detecting short-time micro-vibrations, and while we acknowledge the presence of other types of vibrations, they do not fall within the short time frame we are focusing on. Our focus lies on instances where the monitoring agency is aware of the specific pest it is searching for. For example, in Greece, the primary concern for palms is the red palm weevil, while for mulberries, the focus is on *X. chinenesis*. It is important to note that there may be other co-existing pests, particularly in the case of severe tree damage. Our approach is designed to indicate the presence of woodboring insects as a problem, without specifying the responsible species. The methodology employed in our paper does not aim to differentiate between different and co-existing woodboring species. Instead, the primary objective of this study is to distinguish between the characteristics of a healthy tree and an infested one.

#### Signal Processing

Once the device acquires a new vibrational recording, it proceeds into several signal processing steps to detect and count possible pulses. The case of woodboring insects is a special case in bioacoustics research. The larvae will not disappear shortly after they produce sounds as in the case of vocalizing birds, terrestrial mammals, and cetaceans. Although in the very early instar stages, they do not produce sounds, they will stay in the tree for several months, and during their stay, they will change instar level and inevitably they are going to produce vibrations due to locomotion and feeding activities. Therefore, the first action is to wait for a recording that has low energy (alike to the quiet recordings void of any external noise that we have acquired in the laboratory). If the device acquires a recording that has energy more than a predefined threshold (e.g., 300 times the energy of quiet recordings), the recording is rejected and waits for a quiet ‘clear shot’. If the recordings pass this stage, we extract the envelope of the energy of the signal and we count only vibrational segments that are very short in time (10–20 ms). This is because the fracture of fibers produces micro-vibrations that are inherently brief (between 2–20 ms). Vibrations due to traffic, dog barks, voices, bird vocalizations, etc. have a much longer duration than impulses and thus, they are not counted by the algorithm. The mean energy of the recording and the duration of the vibrational events are the only two features that are used to count impulses. The signal processing part is analyzed in detail below:

The decision about whether a recording contains an impulse can be formulated as a detection problem. Let us consider the following two hypotheses summarized in Equation (1)

**Figure 2:**
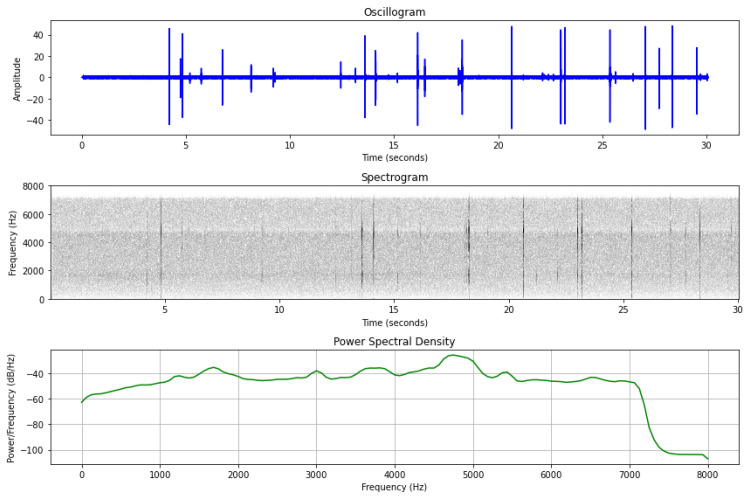
A vibrational recording from an infested mulberry. This is the crack of wooden fibers due to the borer’s activity. (**top**) The vibrational recording, (**middle**) the spectrogram, and (**bottom**) the power spectral density (PSD). Note the ‘train of pulses’ in the oscillogram, the vertical stripes in the spectrogram, and the non-flat distribution of the PSD. Note the short time of each event.

H0: y[n] = s[n]    H1: y[n] = x[n] + s[n], (1)

where y[n] is the digital recording, n is the sample index, x[n] is a pulse or a pulse train due to borers’ activity (see Figure 2), and s[n] is the background vibrational noise. H0 is the null hypothesis and refers to a situation when the observation only includes background noise. In contrast, H1 refers to the situation when an impulse x[n] is observed, in addition to s[n]. The observation interval is n ∈ [0, *N* − 1] where *N* is the length of the signal y[n] in samples. The noise component cannot be assumed to be Gaussian or stationary colored noise as commonly done in other areas of the signal processing literature as it corresponds to the totality of audible vibrations generated by natural sources, animals, and anthropogenic sources. Therefore, s[n] is composed of time-varying, broadband, non-stationary vibrational sources such as birds, dogs, voices, traffic, footsteps, wind, rain, and shaking of leaves that can also vibrate the tree and contribute to y[n]. Depending on the place; urban, forest, or agricultural field, the synthesis of s[n] is different and constitutes the vibroscape (i.e., the vibrational landscape) of the location. The number and the identity of the vibrational sources are a priori unknown. While operating in the field, noise is invariably present in some form. Instances where y[n] = x[n] are rare but can occur, albeit approximately, within specific time windows, primarily during the night. Our objective is to isolate x[n] solely from recordings characterized by quiet conditions and to accurately quantify the micro-impulses within these recordings, distinguishing x[n] from s[n] based on the duration of these impulses. This process is quantified in the following steps.

The spectrogram, a 2D representation of how the frequency power content changes over time, reveals vertical stripes during woodboring activity, indicative of short-time wideband events (see Figure 2(middle)). The power spectral density (PSD) illustrates how the recording’s energy is distributed across its constituent frequencies. In the absence of vibrations, the PSD appears flat, whereas audio events introduce peaks and valleys (see Figure 2(bottom)). Trained observers can distinguish the distinct patterns of time-frequency ‘signatures’ seen as recurrently appearing vertical stripes caused by borers from the vibrations caused by wind or vocalizing animals.

Step 1: Compare the mean energy of the signal against the mean energy of silent signals recorded in the laboratory. Let us denote this Energy per sample *E_sil_*. If the energy of the y[n] signal exceeds a threshold *θ*_1_ (e.g., *θ*_1_ × *E_sil_*), then the recording is rejected. This means that the algorithm waits for silent recordings before it applies counting. The lower the threshold, the more often the sampled vibrations are discarded without further examination, and the decision is postponed until a future silent segment has been received. This results in Equation (2) where *M* is the length of the signal in samples.

(2) $$if\;\sum_{k=0}^{M-1}y^{2}\left[k\right]/M>\theta_{1}\times E_{sil}\;then\;reject\;the\;recording.$$

**Figure 3:**
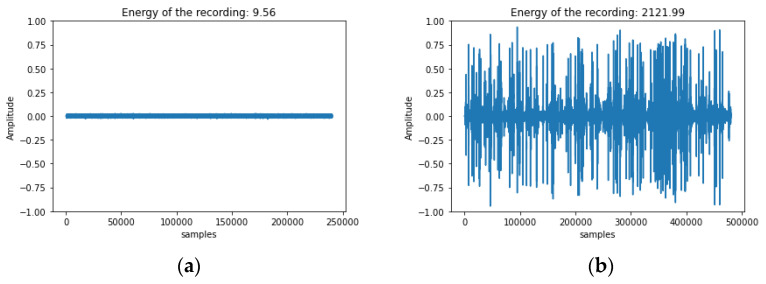
(**a**) A ‘quiet’ vibrational recording from a tree. (**b**) A vibrational recording from the same tree during heavy rain. Note the difference in the energy of the two recordings in the title and how this energy can become a threshold that rejects noisy recordings. The algorithm waits for relatively quiet recordings and only then proceeds into counting vibration events.

In Figure 3, one can see the difference between a quiet signal and a noisy one that has been rejected due to vibrations caused by rain drops hitting the trunk and the device. Counting is not applied to noisy recordings.

Step 2: Convolve the energy of the recording y[n] denoted as y^2^[n] that passes the restriction of Equation (2) with a window w[n] = 1/N for 0 ≤ n ≤ N−1 where N = 200 samples (i.e., apply a moving average filter) to smooth the energy of y[n] and get its envelope in y_sm_[n]. This results into Equation (3) where ⊛ denotes the convolution operation:

(3) $$y_{\mathrm{sm}}[n]=y^{2}[n]⊛w[n].$$

**Figure 4:**
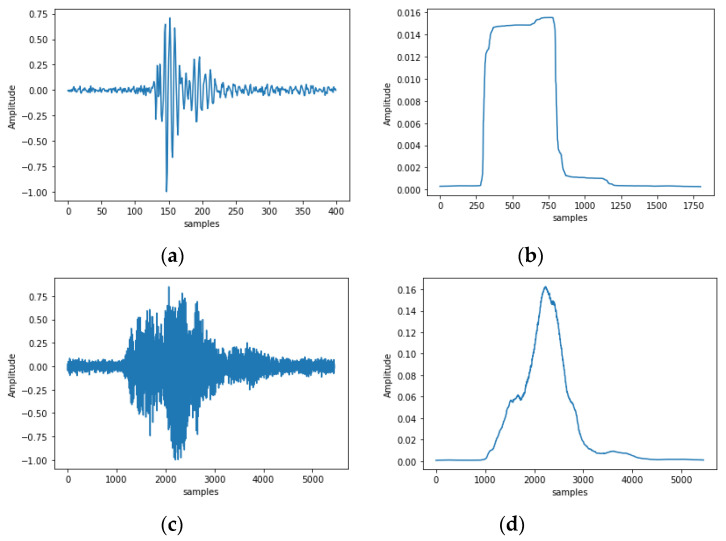
(**a**) A vibrational impulse from a borer in an infested mulberry. (**b**) Smoothing the energy of the signal with a kernel w[n], (**c**) A vibrational impulse from a dog bark, (**d**) Smoothing the energy of the signal of the dog ‘s vocalization with a kernel w[n]. Note the difference in duration between these two vibrational events. The sampling frequency is at 16 kHz.

The Hilbert transform is a mathematical operation that is often used to obtain the analytical representation of a signal. One common application of the Hilbert transform is to extract the envelope of a signal by taking the magnitude of the complex signal and therefore get an equivalent action as of Equation (3). However, for clarity and easiness of implementation and because we did not observe any practical advantage, we continue with (3) as an envelope smoother/extractor instead. A single pulse is illustrated in Figure 4a. If we apply peak-picking directly on the energy signal of a micro vibration, then we may count multiple pulses of the attack-sustain-release phase whereas the event is a single pulse. See Figure 4b to see the effect of extracting the envelope of a borer’s pulse and Figure 4c,d a brief event like a dog’s bark using Equation (3).

Step 3: Partition the energy of the signal is 10 equal segments of *L*/10 samples where *L* is the length of the recording in samples and extract a different noise floor out of the mean value of the energy of each segment. Let ***θ*_2_** be the vector holding the thresholds for all partitions and *c_i_* the number of samples for signal chunk ‘*i*’ and ‘*a*’ is a threshold, then:

(4) $$\theta_{2}=a\times [\frac{1}{C_{1}}\times \sum_{k=0}^{C_{1}-1}{y_{sm}}^{2}\left[k\right],\ldots ,\frac{1}{C_{10}}\times \sum_{k=0}^{C_{10}-1}{y_{sm}}^{2}\left[k\right]].$$

**Figure 5:**
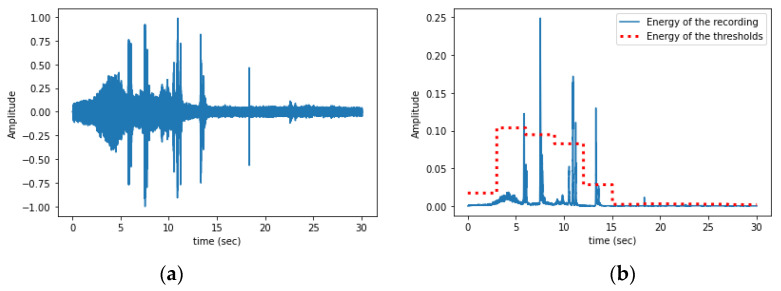
(**a**) A vibrational recording due to the presence of traffic noise and dogs and bird vocalizations. (**b**) The energy of the recording F_20240105121234_12.mp3 with noise levels superimposed. Manual evaluation shows only one valid peak at the 18th s. Note the duration of the valid impulse. Peaks that exceed the threshold are candidates to be included in the counting that will be also screened for their duration before they become valid counts. The algorithm outputs 2 detections.

The number of 10 levels was decided after a grid search on a small subset of the data gathered in laboratory conditions. If an impulse is not present in a segment, then the threshold computation of Equation (3) is exact for this segment. If there is an impulse, then for this segment, Equation (4) is approximate but still close to the true threshold of noise as an impulse has a very short time and does not affect much the computation of the mean. To give an example at 16 kHz, that is the default sampling frequency of the device the length of the chunk is 30 × 16,000/10 = 48,000 samples, and the length of a pulse between 115 and 350 samples (i.e., between 0.2–0.7% of the samples). Practically, Equation (4) is a good approximation of the threshold of noise due to the sparsity of the impulses and their short-time characteristic. If a sample’s energy y_sm_^2^[n], locally exceeds the noise floor for the first time an initiation flag is set to True, and we start counting the duration *D* of the segment. We continue to make a pass on y_sm_[n] until it falls under the threshold of Equation (4), or it reaches its end where the duration calculation of all segments concludes. Depending on if the segment duration denoted as *D* falls inside a permissible short range (i.e., *D*_min_ ≤ *D* ≤ *D*_max,_ where *D*_min_ and *D*_max_ are derived from a subset of high-quality recordings from infested trees), we register the audio event as an impulse and we increase the counter of impulses by one, otherwise we reject the segment. This way we can have reliable counting in the presence of time-varying signals such as traffic noises, strong wind that shakes the branches, rain or dog barks that have significantly longer duration. The counting would fail if a flat level was introduced for the whole recording. See Figure 5 to see the effect of multiple thresholds extracting the envelope of a pulse and of a dog bark. Our tactic is to set a high ‘*a*’ in Equation (4) so that we have a very low rate of false alarms. Even if we have misses because the impulse falls inside a noisy region and is rejected by the threshold **θ_2_**, the larvae produce pulse trains all the time and there will be other pulses in the presence of a more favorable noise background that they will allow the algorithm to detect them in the short run.

## 3. Results

### 3.1. In-Lab Experiments

**Figure 6:**
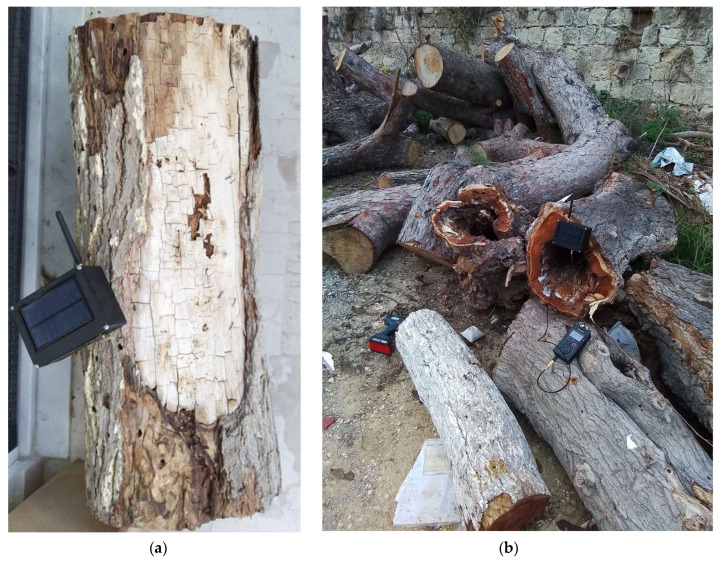
(**a**) Image of a segment of a mulberry tree trunk afflicted by *X. chinensis* infestation. The trunk exhibits exit holes from prior infestations, and the tree displayed apparent signs of degradation prior to being sectioned; (**b**) Collection of predominantly mulberries extracted from urban greenery in the city of Heraklion in Crete, following an ongoing infestation verified to be caused by *X. chinensis*.

Several heavily infested tree trunks of freshly cut trees have been provided by the Municipality of Heraklion (see Figure 6) and have been probed for impulses. We applied the detection approach in Section 2 and parsed the timestamps, and impulses’ characteristics (e.g., impulses’ durations and energies of impulses) in 4747 recordings over a 10-day period from 19 December 2023 to 29 December 2023.

To exemplify the advantages of the automated approach, we present the results of a series of experiments that underscore the complex task of quantifying borer activity, necessitating automated procedures. These experiments are practically unfeasible to conduct manually due to limitations in manpower. Specifically, we investigated the activity patterns of *Xylotrechus chinensis* (Coleoptera: Cerambycidae) within mulberry trunks over the course of a day. *X. chinensis*, an invasive species often identified as a potential threat [12,13], has not been comprehensively studied in terms of its activity patterns throughout the day, the duration of pulses, and the correlation between pulse duration and energy level in decibels, as evidenced by the existing literature. This pest was selected because it extensively infests mulberry trees in the city of Heraklion, Crete, Greece (see Figure 6).

**Figure 7:**
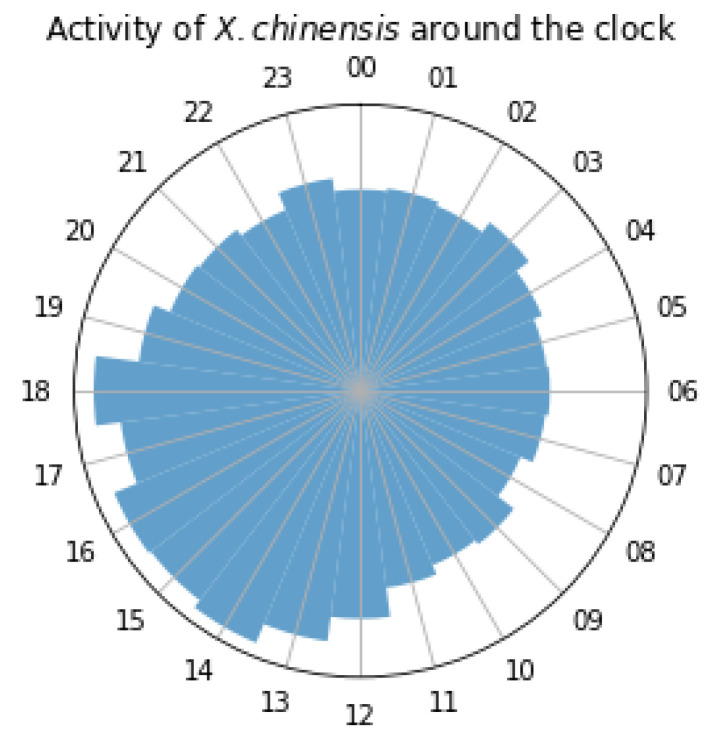
Representation of the hourly activity of woodboring larvae in mulberry tree trunks infested by *X. chinensis*, presented as a circular pie chart. Thousands of impulses from 4747 recordings 30 s each were automatically identified and categorized based on the time of occurrence over a 10-day observation period.

We start this series of experiments with 4747 vibrational recordings that are 30 s long, sampled at 16 kHz being automatically scanned by code realizing Equations (1)–(4). The files and their annotation in terms of counts are open-sourced (see Data Availability Statement). Since these are high-quality recordings without significant background noise, we set *a* = 3 in Equation (4). The impulse detector scanned all files, detected the pulses, and measured several features, such as the total counts per file, the duration of each pulse, and its energy. It produced 56,968 pulses, and these are timestamped upon their creation; therefore, we made a Python code to generate a pie chart depicting activity (peak counts vs. hour of the day). In Figure 7, we see an interesting pattern that arises from grouping almost 57 K pulses according to their hour of appearance: We conclude that woodboring insects are active all day regardless of the hour, but they show increased feeding/tunneling activity during the hours that the sun hits the trunk thus increasing the temperature of the trunk.

**Figure 8:**
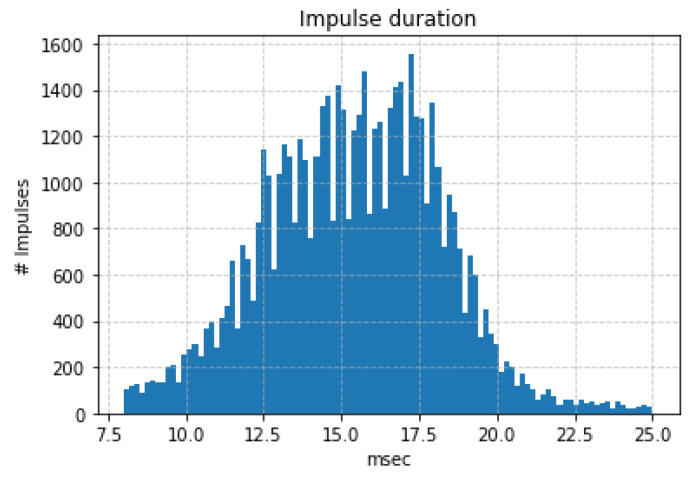
Histogram illustrating the duration distribution of impulses extracted from 4747 recordings generated by the feeding activity of *X. chinensis* in mulberries. The duration of the majority of impulses falls within the 10–20 milliseconds range. It is important to note that different combinations of borers and wood types may yield different durations. Note that the exact number and locations of larvae within the trunks remain unknown and the durations prior to the application of the window w[n] are smaller (i.e., the true duration is 2–12 ms).

In Figure 8, we derive a histogram of the duration of all impulses which is found to be between 10–20 ms with the most probable duration at 16 ms. 

**Figure 9:**
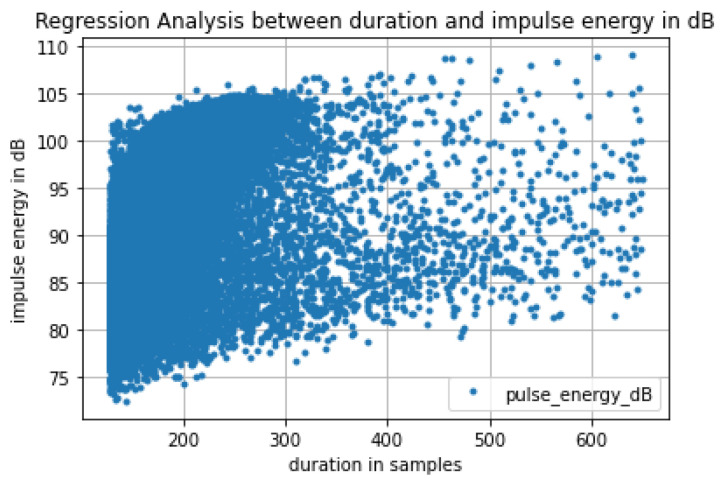
Scatterplot detailing the energy in decibels (dB) of signal chunks corresponding to impulses versus duration. The graphic emphasizes the uncertainty surrounding the exact number and locations of larvae within the trunks, and their potential distance from the probe, which could contribute to the observed variations in energy levels.

In Figure 9, we quantify the relationship between impulse duration and energy of an impulse in dB. A scatterplot shows this relationship. The fact that for a specific duration in the range 150–400 samples at 16 kHz the impulse energy is spread out to a range of amplitudes indicates the existence of multiple larvae that are located at different distances from the probe.

**Figure 10:**
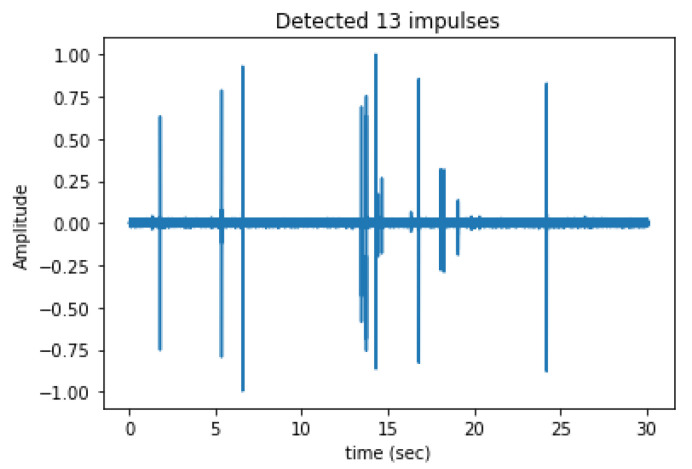
A characteristic example of infestation and correct counting of impulses.

The proposed approach facilitates the automated monitoring of trees to detect potential infestations and assess their severity based on both the quantity and rate of impulses per minute. While the algorithm incorporates two thresholds to minimize false alarms, it cannot entirely discard vibrations originating from sources external to the tree. However, it effectively ignores a significant portion of these extraneous vibrations, such as those resulting from speech, animal vocalizations, wind, and traffic. There is always an inherent tradeoff between misses and false alarms in any detection problem, regardless of its application domain. In our task, the false alarms are due to vibrations induced to the tree by external vibrational sources. In operational conditions, the priority is to suppress false alarms that will misguide us to classify a tree as infested. In the bioacoustics case of detecting woodboring insects inside their host, we have the luxury to set a strict acceptance threshold leading to an operational point that will significantly reduce false alarms but, inevitably, will also increase misses. In the context of this application, it is tolerable because the borers will repeatedly produce vibrations for a long time (months) and their perpetual activity will compensate for the miss of impulses if they indeed are there. Therefore, it is better to reject a noisy signal (even if it contains useful borers’ impulses) and wait for a ‘clear shot’, meaning for a more favorable combination of impulses and background noise. To give a lucid example, even if the algorithm discards 99 out of 100 recordings due to noise and it gets a single recording like the one in Figure 10, this is enough to classify the tree as infested. In Figure 10, we see a clear impulse train with many brief impulses that gives in irrevocably the situation of the tree. If the tree is indeed infested and depending on how frequently we have set the sampling intervals, we may get this signal in one to several days. If the tree is not infested, there is no reason to ever get a sequence of events like the one in Figure 10.

**Figure 11:**
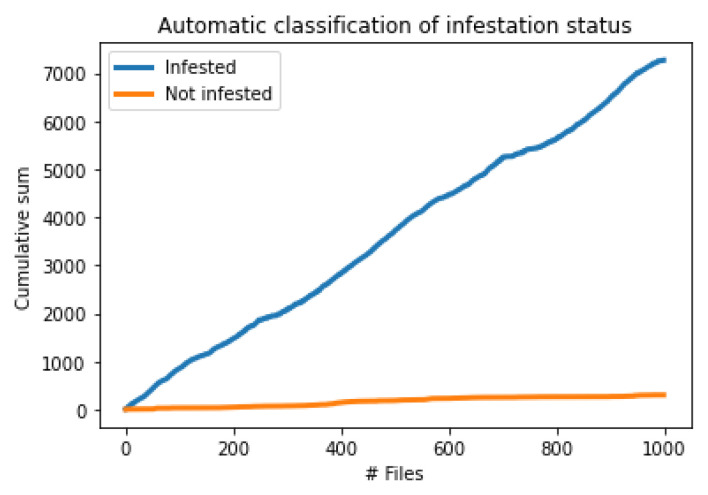
A plot on cumulative impulse counts from 1000 files of 30 s duration coming from infested trunks (top blue line) and recordings that contain only environmental vibrations (i.e., not infested) in the bottom orange line.

We followed the approach of strict thresholds that oppress the false alarms, and we performed another experiment where we compared the results on two folders one coming from an infested tree and one from inserting the probe into the ground in an urban environment. We set a strict θ_1_ = 185 and *a* = 10 and we run the algorithm on 1000 files from each recording and we monitor in Figure 11 the cumulative sum of impulses as time goes by.

It is essential to highlight that the infested tree case yields beyond doubt a significantly larger number of impulses compared to the non-infested folder. Figure 11 is characteristic of what the user sees when one probes a tree. Nevertheless, the potential for obtaining inaccurate counts from non-infested cases underscores the necessity to prolong the monitoring duration and supplement it with various forms of evidence to accompany event counts per file, such as vibrational records. Inspection of the oscillogram, spectrogram, and power spectral density (PSD) analyses provide additional information. It is important to emphasize that a trained operator possesses the capability to discern and discard almost all false cases by actively listening to the recorded data.

### 3.2. Field Experiments

We assessed the automated procedure in an alley within the city of Heraklion to identify infestations in living mulberries. In the first experiment, we installed the device and monitored a single tree for about 1 week. The probe was inserted in the point where the branches split in the beginning of the crown. We manually tagged the 596 files that were transmitted from the tree straight to the server in terms of number of impulses and annotated the background noise type. Manual annotation allows to compute exact error metrics in Equations (5) and (6). Mean absolute error (MAE) is a measure of errors between paired values expressing the same phenomenon. MAE is the arithmetic average of the absolute errors where *y_i_* is the prediction of the total number of impulses in file ‘*i*’ and *x_i_* the true counts as defined by the human annotator.

(5) $$MAE=\sum_{i=1}^{n}\frac{\left|y_{i}-x_{i}\right|}{n}.$$

The root-mean-square error (RMSE) is the quadratic mean of the differences between the observed values *x_i_* and predicted ones *y_i_*.

(6) $$RMSE=\sqrt{\sum_{i=1}^{n}\frac{{\left(y_{i}-x_{i}\right)}^{2}}{n}}.$$

The outcomes are presented in Table 1. The vibrational recordings and the corresponding annotation file are open source. In the Data Availability Statement, one will find other trees as well that have been examined using a smaller time span of 1–15 min.

We wanted to assess the impact of rejecting noisy recordings against applying impulse counting to all recordings. In Table 1, we see that avoiding doing so, almost doubles the error metrics and because RMSE is sensitive to large divergences the approach of examining all recordings can give large errors (e.g., in the case of heavy rains, or when branches are shaking due to strong winds). The error metrics show a very satisfying performance which entails that the impulses produced by xylophagous insects are shorter in time than other brief vibrational sources such as dog barks, hits, and footsteps. The energy envelope of other competing noise sources such as voices, traffic has a longer duration that makes it easy to reject them. The noisy recordings due to rain need to be discarded based on the total energy of the recording as raindrops can be very close to the brevity of borers’ impulses (see Figure 3b and the energy of the recording in the title compared to the energy of a very quiet recording in Figure 3a). 

**Figure 12:**
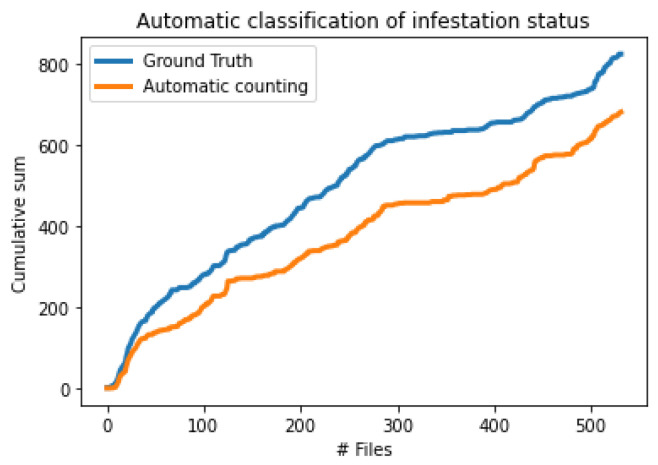
Automatic classification of the infestation status of a living mulberry in the field. Data have been gathered over a week’s period. Plot of the cumulative impulse counts from 596 files of 30 s duration transmitted via the mobile network from the tree. The top blue line is the ground truth annotated manually whereas the bottom line is the result of automatic counting. The fact that the lines are not forming plateaus almost parallel to the *x*-axis indicates that the tree is infested. The inclination of the lines categorizes the severity of the infestation. Detailed annotation of the recordings can be found in the Data Availability Statement.

**Figure 13:**
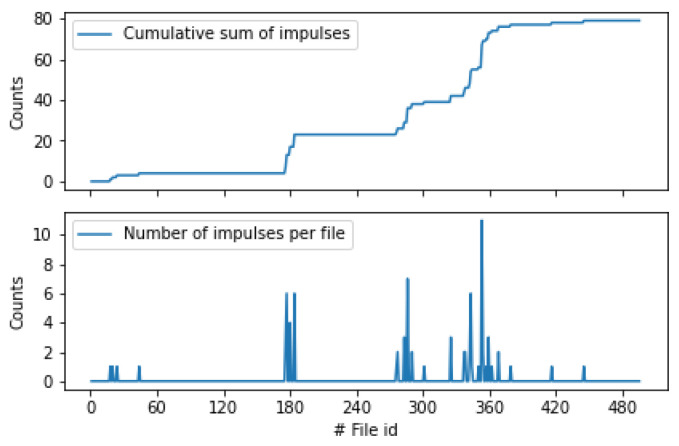
Automatic classification of the infestation status of a healthy fig tree (*Ficus carica*). Data have been gathered over a three-day period with intensive sampling. All detections are false alarms because the tree is healthy. Note the plateau of the cumulative graph with the *x*-axis indicating a very slow gradual increase in total pulses.

In Figure 12, we visualize the results between ground truth (i.e., manual annotation of data) and automatic impulse counting. One can see that the system follows closely the correct data. In Figure 13, we repeat the same for a healthy, young fig tree with the same settings/thresholds as in Figure 12. The interested viewer may compare Figure 13 (healthy) against Figure 12 (infested). Figure 13 is characterized by successive extensive plateaus and recordings with mostly a single impulse and not clusters of impulses. 

The focus of this study is on classifying a tree after prolonged monitoring. In this regard, all datasets in the test folder have been correctly classified as originating from infested trees (see Data Availability Statement for data folders and close pictures of the trees). While classification at the recording level is feasible, it does not align with the core principles of our approach. Our methodology involves counting only the quiet recordings and disregarding many that may contain useful impulses. However, to address evaluation transparently, we also calculate accuracy, F1-score, precision, and recall on a per-recording basis for the 7-day period of a single mulberry tree (see Table 2).

Precision (*P*) is defined as the number of true positives (*T_p_*) over the number of true positives plus the number of false positives (*F_p_*). Recall (*R*) is defined as the number of true positives (*T_p_*) over the number of true positives plus the number of false negatives (*F_n_*). The *F*1 score is defined as the harmonic mean of precision and recall. We can compute the *F*1 using the following Equation (7):

(7) $$P=\frac{T_{p}}{T_{p}+F_{p}},\;R=\frac{T_{p}}{T_{p}+F_{n}},\;F1=\frac{2P*R}{P+R}$$

Regarding power consumption, the device delivered 596 recordings of 30 s each, each were sampled every 15 min and were gradually transmitted every 2 h for a period of one week and lost 3% of its power. As the leaves of the trees were removed, the energy harvesting capability of the embedded solar panel had a direct view of the sky and this delivered 2% of charging capacity each day on sunny winter days and 0.5–1% on cloudy days. Depending on how frequently we sample the internal vibroscape of the tree and how often we deliver data, the device can function, as a rule of thumb, from 2 months–1 year without manual recharging. If the weather conditions are favorable, the device should work permanently without any need for manual recharging.

## 4. Discussion

**Figure 14:**
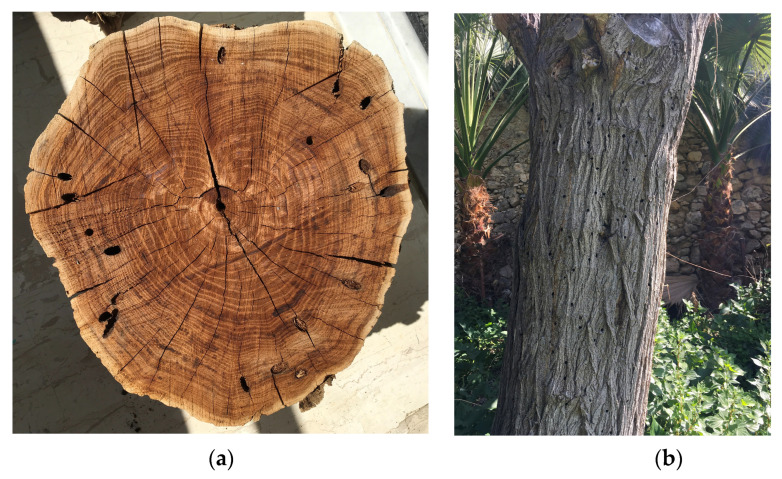
(**a**) A 38 cm diameter piece of trunk was used for some of the in-lab experiments. The holes close to the circumference are tunnels the larvae dug. The probe was inserted in the center. (**b**) In living trees, it is not easy to locate the larvae though one can see the signs left from the activity of previous generations (notice the exit tunnels on the trunk).

We envision a connected world where trees are extensively linked to the internet, allowing for the automatic monitoring of various parameters related to their health, including their infestation status with xylophagous pests. We have successfully addressed challenging technical obstacles to present for the first time, to the best of our knowledge, a device at a high Technological Readiness Level (TRL-9), capable of automatically detecting woodboring insect infestations in trees. This novel device operates by counting impulses generated from fiber cracking, induced by the feeding and locomotion activities of larvae residing under the bark and/or within the trunk. The algorithm, coded in C, is integrated into the device, enabling real-time signal analysis and decision-making on-site. Additionally, the severity of infestation can be assessed based on the rate of occurrence of impulses recorded. Furthermore, if the tree undergoes biological treatment with injected insecticides or fungicides, the device can implicitly evaluate treatment efficacy by monitoring reductions in impulse rates [3]. The determination of the infestation status is crucial, given the uncertainty of larvae presence within the trunk and their specific locations (refer to Figure 14). 

The further the larvae are from the probe, the smaller the signal-to-noise ratio becomes. The lower the instar level and the fewer the number of larvae, the sparser the train of impulses becomes, resulting in a lower signal-to-noise ratio as depicted in Figure 10. In trees with dense, solid wood, the detection radius depends on the size of the pest but generally ranges from 1 to 2 m. Phytosanitary personnel are typically aware of the anticipated location of infestation and should position the probe accordingly. However, for plants with tall, slender, fibrous trunks like palms, the detection radius may be smaller, necessitating the placement of the device at the crown. Conversely, in many other trees such as mulberries, the probe should be positioned at the branch split.

Practical observations: (a) In urban environments, the device needs to be camouflaged and attached to a sufficient height to avoid theft or vandalism. In a forest, with a plantation or an agricultural regime, this restriction can be relaxed as the device is almost cryptic. (b) The electronic board has a bilateral communication with the server through the mobile network, i.e., it uploads data and receives commands (firmware updates, time scheduling of actions, reconfiguration of recording durations, and delivery of data among other tasks). Therefore, it can be configured to transmit only counts, GPS, coordinates, telemetry, and environmental variables solely, an action that substantially prolongs power sufficiency. However, we have found it useful to operate it in a way that delivers an mp3 compressed recording as well when the counter of the detector has a positive value. This way it screens a large number of recordings that have zero counts of impulses before directing only the interesting ones as evidence to a human observer that may listen to the tree, increase the duration of future examinations if one feels so and have a look at the spectrogram and the PSD of the vibrations. Scrutinizing the power spectral density (PSD) and spectrogram of the recordings has an advantage over auditory assessment and this lies in the rapid interpretation facilitated by the human eye and brain. The PSD and the spectrogram visualize sound and visual interpretation can supplement or replace auditory evaluations, making it feasible to analyze the recordings faster (see Figure 2). (c) Decisions should not rely solely on a single impulse, as it could stem from timber or trunk dilations or an impact on the trunk. A consistent pattern of repeatable impulse trains must be observed across multiple recordings, followed by manual verification, before reaching a final decision. While the device can still encounter errors, particularly from very brief external vibrational sources like light rain or brief bird calls, the observer must independently review the evidence by listening. Nonetheless, the device substantially reduces the effort required and provides supporting evidence for more informed decision-making.

Irrespective of the wood type and the borer species, the count of very short-time impulses within a preconfigured time span serves as a universal ‘feature’ for detecting feeding/tunneling activity inside the tree trunk or beneath the bark. Distinct combinations of wood substrates and insects result in impulses with varying spectral content, duration, and energy. Exploring the possibility of further classification to identify insect species, estimate their density, or even localize them within the trunk holds considerable potential. 

Comprehensive information is provided on a repository of vibrational recordings along with their automatically generated annotation files (refer to the Data Availability Statement for details). This database serves as a resource for researchers interested in exploring innovative methods for insect detection and severity assessment concerning woodboring insect infestations. By leveraging this extensive database derived from a single insect species and mulberry trees (*Morus* sp.), one can access impulses and extract embeddings using diverse deep learning models as first suggested in [14] and followed in [15,16]. These embeddings facilitate the adaptation of the models of different deep-learning classifiers to various combinations of wooden substrates and woodboring insect species with minimal sample requirements (i.e., few-shot learning and classification). The effectiveness of this approach, coupled with its successful application in innovative bioacoustics tasks, has been demonstrated in prior research focused on birdsong classification [17]. Therefore, a future direction is to have our approach—which is very fast and very low in power consumption—do the heavy screening of searching for needles in the hay and direct only a small subset to heavy deep-learning models that will scrutinize these cases especially when one applies low thresholds (that produce false alarms) with the aim to detect early instars and low infestation load [18,19,20].

Automatic monitoring of trees produces a continuous, almost real-time, stream of data that can be directed to a DSS that monitors trees at a large scale and communicates actions and messaging without human intervention. Our approach allows detection methods leveraging artificial intelligence to establish an early warning network at a country/continental level and new intervention schemes, with the potential for expansion to address other wood-boring insects beyond the specified study case.

Last, in the near future, this stream of data can be managed directly by large language models such as ChatGPT with cooperating plugins such as signal analysis that can fuse information from a dispersed network of probes with weather data and epidemic models to visualize answers on questions that are posed to the system in natural speech [2]: Is the tree infested? Where is the problem in my plantation and how serious is it? How is this condition treated or managed? How can I learn more about my pants condition? How will it probably evolve?

## 5. Conclusions

The primary purpose of the remote vibroacoustic approach is the extended and systematic monitoring of trees and wooden structures to guard against borers. Automatic monitoring of trees for wood-boring insects represents, in our point of view, a significant advancement, enabling the practical monitoring of an extensive number of trees. Leveraging IoT communication and global SIM cards further allows for the remote monitoring of trees globally. However, challenges arise when dealing with thousands of recordings from hundreds of trees. In such cases, automated procedures, such as the one presented in this work, become essential to significantly reduce the need for human observation in determining whether a tree is infested. Remote control, energy harvesting, and its low-power implementation reduce the necessity for maintenance. The decision-making is based on applying strict thresholds on the energy of the recording so as to discard noisy ones (e.g., wait for quiet, favorable conditions) with emphasis on reducing false alarms even if misses are increased and then counting only the impulses that have small duration.

## Figures and Tables

**Table 1:** Evaluation of automated counting of impulses against the true number of impulses on 596, 30 s files transmitted from a single tree over a week, operating 24/7.

Approach	MAE	RMSE	Parameters
Reject noisy recs, evaluate on all recs	1.07	2.00	θ_1_ > 185, *α* = 7 (Equations (3) and (4))
Do not reject recs, evaluate on all recs	1.71	4.93	*α* = 10
Evaluate only accepted recs	1.01	1.91	θ_1_ > 185, *α* = 7 (Equations (3) and (4))

**Table 2:** Binary classification (infested/not-infested) error metrics on per recording basis for 532 out of 596 30-second recordings (64 files discarded as too noisy based on Equation (2)).

	Precision	Recall	F1-Score	Support
No pulses	0.68	0.83	0.75	236
Pulses	0.84	0.69	0.76	296
accuracy			0.75	532
Macro avg	0.76	0.76	0.75	532
Micro avg	0.77	0.75	0.75	532

## Data Availability

All data refer to the pest *X. chinensis* in different mulberries. We have uploaded the following folders to the open repository ZENODO: https://zenodo.org/uploads/10820310 (https://doi.org/10.5281/zenodo.10820310) (accessed on 19 April 2024) the following folders: (1) ‘Infested’. Contains 4747, 30 s vibrational recordings sampled at 16 kHz in mp3 format. They are high-quality recordings from various infested pieces of mulberry trunks. The recordings have been delivered through the WiFi functionality. The ‘infested_folder_automatic.csv’ is a csv file that contains automated annotation results for the ‘infested’ folder in terms of pulses. The infested_folder_automatic_detailed.txt’ includes location, duration and energy, and precise timestamp in local hour (Athens time-zone). (2) ‘Not Infested’. Contains 1091, 30 s vibrational recordings sampled at 16 kHz in mp3 format. They are high-quality recordings from background noise only. The probe was inserted into the ground. The recordings have been delivered through the WiFi functionality. (3) ‘Not Infested fig tree’. Contains 500, 30 s vibrational recordings sampled at 16 kHz in mp3 format. They are high-quality recordings stemming from a healthy fig. The probe was inserted in the trunk. The recordings have been delivered through the WiFi functionality. (4) ‘test_set’. Contains the subfolders: infested_1, infested_2, infested_3, infested_4, infested_5. They all contain recordings taken with a TASCAM DR-40X portable recorder that received signal through the line-out socket of the proposed device. All recordings have been down sampled to 16 kHz and compressed to mp3. Each subfolder belongs to a different living tree. While these five mulberry trees are not aged, they exhibit some signs of infestation, albeit not to the extent observed in older trees. As of 5 January 2024, there are no immediate plans for the removal of these trees in the foreseeable future. Determining the infestation status of a tree requires prolonged monitoring (several days to weeks), but practical constraints, such as concerns about theft and vandalism of exposed devices in open spaces, limited our monitoring to 15 min per tree. (5) mulberry_7_days, contains 596, 30 s vibrational recordings sampled at 16 kHz in wav format, originating from a single tree monitored on a 24/7 basis for a week. This folder is manually annotated in terms of background noise and how many impulses a trained observer has attributed per file (mullbery_7_days.xls). The subfolder there is another folder called ‘mulberry_7_days_visual_automatic_annotation’. This folder has detailed pictures of the oscillogram, the energy signal of each recording with the energy thresholds super-imposed, where the detector thinks the impulses are, the detected number of impulses, and the ground truth in terms of counted impulses. The structure of the records is a list of files like the following: F_20230812193118_1.mp3. F stands for File, 2023 stands for the year, 08 for the month (August), 12 for the day of the month, 19 for the hour of the day, 31 for the minute of the hour, and 18 for the second of the minute (therefore the time stamp is 12 August 2023 19:31:18). The symbol _1 is an integer number associated with consecutive recordings. Code that processes the data can be found at https://github.com/potamitis123/TreeVibe.v3 (accessed on 19 April 2024).
